# Modifications of Nanobubble Therapy for Cancer Treatment

**DOI:** 10.3390/ijms25137292

**Published:** 2024-07-02

**Authors:** Katarzyna M. Terlikowska, Bozena Dobrzycka, Slawomir J. Terlikowski

**Affiliations:** 1 Department of Food Biotechnology, Medical University of Bialystok, Szpitalna 37 Street, 15-295 Bialystok, Poland; katarzyna.terlikowska@umb.edu.pl; 2 Department of Gynaecology and Practical Obstetrics, Medical University of Bialystok, M. Sklodowskiej-Curie 24A Street, 15-089 Bialystok, Poland; bozena.dobrzycka@umb.edu.pl; 3 Department of Obstetrics, Gynaecology and Maternity Care, Medical University of Bialystok, Szpitalna 37 Street, 15-295 Bialystok, Poland

**Keywords:** nanobubbles, cancer, doxorubicin, paclitaxel, low-intensity ultrasound, hematoporphyrin monomethyl ether, lonidamine, siRN, photodynamic therapy, sonodynamic therapy

## Abstract

Cancer development is related to genetic mutations in primary cells, where 5–10% of all cancers are derived from acquired genetic defects, most of which are a consequence of the environment and lifestyle. As it turns out, over half of cancer deaths are due to the generation of drug resistance. The local delivery of chemotherapeutic drugs may reduce their toxicity by increasing their therapeutic dose at targeted sites and by decreasing the plasma levels of circulating drugs. Nanobubbles have attracted much attention as an effective drug distribution system due to their non-invasiveness and targetability. This review aims to present the characteristics of nanobubble systems and their efficacy within the biomedical field with special emphasis on cancer treatment. In vivo and in vitro studies on cancer confirm nanobubbles’ ability and good blood capillary perfusion; however, there is a need to define their safety and side effects in clinical trials.

## 1. Introduction

The development of cancer is associated with genetic mutations in primary cells. It is estimated that between 5% and 10% of all cancers are derived from acquired genetic defects, with the majority of these being a consequence of environmental and lifestyle factors [1]. In 2023, the total number of cancer-related deaths in the five largest countries in Europe was predicted to be 1,261,990 (702,214 men and 559,776 women). The corresponding age-specific rates (ASRs) are 123.8/100,000 for men and 79.3 for women, representing a decrease of −6.5% and −3.7%, respectively, compared to the rates observed in 2018. The statistical projections indicate a favorable outlook for all cancer sites considered and both sexes, with the exception of lung and pancreas among women. The ASRs for these two sites have increased compared to 2018, with the ASR for pancreas rising by 3.4% and the ASR for lung cancer by 1.2% [2]. Overall, the downward trend is consoling, but the cancer death numbers are still too high.

In the 1950s, Dr. Otto Warburg was the first to report that cancer cells acquire metabolic alterations compared to normal cells [3]. In the tumor microenvironment, cancer cells adopt their metabolism to unfavorable conditions, e.g., hypoxia and acidic pH caused by a lack of microcapillaries and the induction of glycolysis (Warburg effect) mediated through hypoxia-inducible factor (HIF1α). This process is accompanied by lactate and H+ extracellular efflux [4]. In addition to glycolysis, recent studies have also focused on the metabolism of lipids, one-carbon, acetate, and amino acids in cancer. The evidence clearly indicates that essential amino acids like leucine and valine play a crucial role in promoting cancer progression through the mTOR complex. Additionally, these amino acids collaborate with the stress adaptation mechanisms of HIF1α, sterol regulatory element-binding protein 2 (SREBP2), and activating transcription factor 4 (ATF4) to further enhance their impact [5]. Previous assumptions held that amino acid metabolism through the mTOR pathway constituted the primary signaling cascade complex. However, mTOR inhibitors such as rapamycin have been demonstrated to be incomplete cures for cancer, suggesting the existence of an adaptive amino acid metabolism that is not mediated by the mTOR complex [6]. Metabolic signaling by glucose, fatty acids, and amino acids has been found to differ between cell–cell and organelle–organelle interactions. A more detailed understanding of cancer metabolism could help in understanding cancer pathogenesis. Several factors lead to increased cancer malignancy, later invasion, and potential metastasis, such as hypoxia, poor nutrition, and an acidic pH resulting in metabolic alterations [7]. 

Also, drug resistance causes more than half of cancer-related deaths [8]. Tumor cell drug resistance is caused by a variety of mechanisms, including increased multidrug resistance (MDR) during long-term chemotherapy, improved DNA repair ability, genetic mutation, heterogeneous biological metabolism, apoptosis pathway obstruction, microenvironmental changes, and drug target changes [9]. Each of the aforementioned mechanisms reduces drug efficacy while increasing the difficulty of tumor treatment. The interaction of genes and signal regulatory pathways makes it impossible to provide a straightforward explanation for tumor MDR. As research on the transcriptome and proteome continues to advance, the regulatory mechanisms of multidrug resistance (MDR) can be elucidated in a multi-dimensional manner, providing a reference for the clinical reversal of tumor resistance [10].

Chemotherapeutic drugs, on the other hand, have limited efficacy due to side effects such as systemic toxicity [11]. The local delivery of chemotherapeutic drugs may reduce toxicity by increasing therapeutic dose at targeted sites while decreasing plasma levels of circulating drugs. Nanobubbles have sparked widespread interest as a potential drug delivery system, owing to their non-invasive and targeted nature. Nanosized particles cross the capillary wall more efficiently than traditional microbubbles and can be transported to the target site without causing any harm [12]. The purpose of this review is to present the characteristics of nanobubble systems and their efficacy in the biomedical field, with a focus on cancer treatment. 

## 2. Nanobubble Characteristics

The International Organization for Standardization (ISO) defines a nanobubble as a gaseous domain enclosed in a medium with a size ideally below 1 µm [13]. Nanobubbles, given their stability and long lifetime, achieved universal industry applications, for example, in food and drinks [14], household chemicals [15], the removal of rust [16], waste management [17], hydroponics [18], fossil fuels [19], and medical production [20]. Surface nanobubbles (SNBs) are defined as bubbles that can be attached to an immersed surface or trapped in a crevice. Bulk nanobubbles (BNBs) are dispersed throughout the bulk liquid. The phenomenon of nanobubbles was first described in the early 1990s by Parker and colleagues, who conducted experiments to investigate the interactions between two hydrophobic surfaces immersed in water [21]. The atomic force microscopy (AFM) device allows the visualization of very small SNBs up to 100 nm [22].

Nanobubbles can be created using various techniques, such as solvent exchange, gas purging, or oversaturation. The creation of BNBs is done through mechanical methods (agitation, vortexing, sonication, compression, decompression, hydrodynamic cavitation, and microfluidics). In general, mechanical methods, such as micro/nanobubble generators, are employed for aeration. These methods typically result in the further expansion and subsequent exit of the system through buoyancy. In contrast, the remaining processes result in the shrinkage and collapse of the system, which ultimately leads to the formation of nanobubbles [23]. 

The primary methods for characterizing BNBs are dynamic light scattering and nanoparticle tracking analysis. Both techniques rely on light scattering and are intended to measure nanoparticle sizes. The resolution is approximately 1 nm, and it is not limited by the Abbe limit (a microscope’s diffraction limit) [24]. 

## 3. Construction of Nanobubbles in Biomedicine

**Figure 1:**
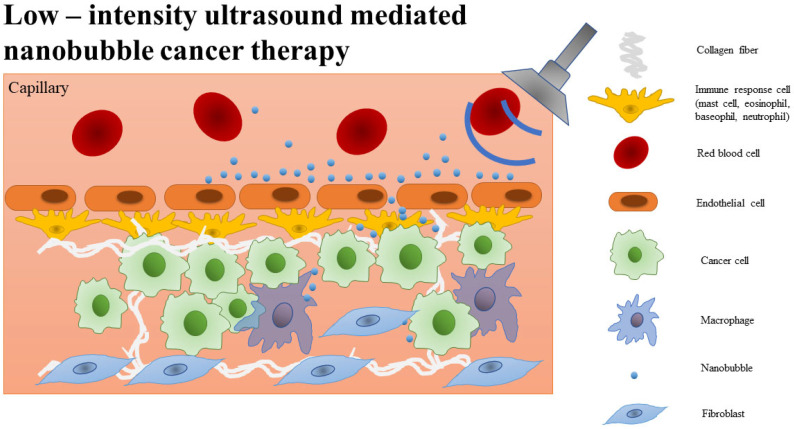
Low-intensity ultrasound-mediated nanobubble cancer therapy.

Narrow particle size ranges and their low size distributions influence pharmacokinetics, uptake mechanism, distribution, and clearance in the human body [25]. Thus, the small size of nanobubbles allows for extravasation and safe passage through blood capillaries and the blood-brain barrier (BBB) [26]. Covering the nanobubble surface and filling the core with various compounds is possible. By slowing down the pace of gas diffusion, the shell keeps the bubble core’s elasticity, stability, half-life, and protection intact by preventing disproportionation, coalescence, and excretion [27]. The in situ incorporation of multiple gases within the core enhances overall stability, with one type of gas aiding the binding of another. Ultrasonication is commonly used to transport nanobubbles because the gas within the core acts as a harmonic oscillator. The bubbles’ reaction is determined by the intensity of the ultrasonication (Figure 1). 

It has been demonstrated that low-intensity ultrasound can direct nanobubbles to a targeted area, while high-intensity ultrasound causes the bubbles to collapse. This collapse leads to a localized increase in membrane permeability, enabling exogenous molecules to enter the cells or tissue. The targeting of nanobubbles with ultrasound initiates a series of mechanisms, including cavitation, sonoporation, acoustic radiation force, acoustic streaming, and finally, a thermal effect. The oscillation intensity is dependent on the acoustic intensity, which can be classified as either low or high. Low intensity oscillations are characterised by stable movement, whereas high intensity oscillations exhibit a non-linear, asymmetric manner with explosive growth and bubble collapse. The oscillation of the bubbles generates micropores in the cell membrane, a phenomenon known as sonoporation. The acoustic radiation force exerts a directional force on the particles, leading to the aggregation and dispersion of the bubbles. The mechanical friction of particles replaces mechanical forces with heat energy. Acoustic streaming is a steady flow in a fluid driven by the absorption of high-amplitude acoustic oscillations, which together with heat, helps to facilitate the migration of bubbles and their cargo through endocytosis [28]. 

The shells of nanobubbles can be constructed of polymers, surfactants, phospholipids, lipids, and proteins and are classified as either hard or soft [29]. Other factors that can influence the stability of a bubble include a negative charge on the surface, hydrogen bonding between the bubble and solution, and contaminants at the gas–liquid interface [30]. The modification of the lipid chain length, modification of its chemical structure, or addition of polymers also reflect on bubble durability. The most popular polymers are poly(lactic acid) (PLA) and poly(lactic-*co*-glycolic acid) (PLGA). These polymers are neutral, biodegradable, and they increase the bioavailability of nanobubbles. Extracellular molecule accumulation is reduced, thus avoiding possible adverse effects [31]. Phospholipids are common shell material as their preparation is simple and offers both biocompatibility and biodegradability. Lipid shells are used for their drug loading and acoustic properties [30]. Nanobubbles with liposome shells are a popular choice due to their ease of modification with conjugates [32]. Surfactants are indispensable components of the shell, contributing to the stability of BNBs by reducing surface tension. Polyethylene glycol (PEG), an approved surfactant, enhances biocompatibility and may assist in reducing immunogenicity due to its biologically inert nature. Furthermore, PEG enhances the stability and half-life of the bonded material [33]. Additional modifications include the utilization of antibodies to enhance nanobubble targeting. Nanobubbles constructed from hydrogel shells have been subjected to testing for the purposes of drug and gene delivery. Hydrogels are both flexible and hydrophilic, preventing gas dissipation, which is controlled by varying the degree of hydrogel swelling, and improving echogenicity due to their flexibility [34].

**Figure 2:**
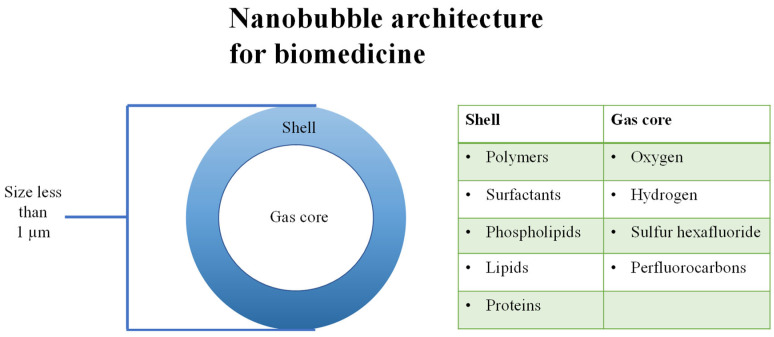
Nanobubble architecture for biomedicine.

The stability of the bubble is maintained by the gas core, the surrounding solvent, and the shell. The specific type of gas must be carefully considered in relation to the intended purpose [35]. Ion-stabilized gas nanobubbles are most dense in gases with dipole moments [36]. The nanobubble cores are predominantly formed of hydrophobic and inert gases with low solubility, resulting in a slower dissolution rate [37]. Gases used in nanobubbles for biomedical applications include oxygen [38], hydrogen [39], sulfur hexafluoride [40], and perfluorocarbons, including octafluoropropane, perfluorobutane, and perfluoropentane [41]. Perfluorocarbons are frequently used as they have a low solubility in an aqueous phase and are inert and not toxic to normal human cells [42]. 2H, 3H-decafluoropentane is a liquid at body temperature and is used to generate nanodroplets and nanobubbles [43]. The gases within the bubbles have both medicinal and ultrasonic imaging precision-enhancing properties. Enclosing multiple types of gas within a bubble in order to enhance the stability effect is also possible [44]. Since perfluoropentane encourages oxygen trapping, the co-encapsulation of the gases inhibits spontaneous oxygen release. Figure 2 depicts the structure of a nanobubble.

## 4. Nanobubble Modifications against Cancer

Although the long-term existence of nanobubbles (range of weeks to months) is still not fully understood [45], the medical field has benefited in recent years from the versatile applications of nanobubble technology as ultrasound contrast agents and treatment tools. They are being used as theranostics agents, and in ultrasound-triggered medication administration and imaging [46], targeted gene delivery [47], skin disease treatment [48], oxygen delivery to hypoxic tumors [49], and neurological illnesses, to mention a few [50]. Nanobubbles are different from microbubbles because they can enter regions that are difficult to reach with other treatments. This is mainly due to possible modifications, size, maintaining stability, and the surface area to volume ratio (S/V ratio).

Doxorubicin is in the anthracycline and anti-tumor antibiotic family of medications [51]. It partly functions by disrupting DNA’s ability to function. Doxorubicin interacts with DNA in two distinct ways: it intercalates and inhibits macromolecular production. It prevents topoisomerase II from progressing, which is an enzyme that relaxes supercoils in DNA to allow transcription. Doxorubicin stabilizes the topoisomerase II complex after it has broken the DNA chain for replication, preventing the DNA double helix from being released and therefore terminating the replication process. Moreover, it may enhance the generation of quinone-type free radicals, adding to its cytotoxicity [52]. Chitosan, a polysaccharide, has sparked widespread interest due to its natural origin, biodegradability, biocompatibility, antibacterial activity, and low immunogenicity. Chitosan is an N-deacetylated chitin derivative, one of the most common biological components occurring in nature [53]. In the study of Zhou et al. [54], doxorubicin was loaded into chitosan nanobubbles and applied with ultrasound to breast cancer cells in vitro. Chitosan nanobubbles with a perfluoropropane core and doxorubicin hydrochloride combined with ultrasound significantly increased the percentage of apoptotic cells compared to the group with free DOX. The outcomes of this study and others mentioned below are shown in Table 1. 

Tumors in low-oxygen environments are more resistant to chemotherapy, radiation, and photodynamic therapy [55]. Hypoxic conditions stabilize a protein called HIF-1α, which has been linked to tumor resistance to these treatments [56]. HIF-1α in tumor cells causes them to switch to anaerobic metabolism and makes them express several genes involved in angiogenesis, apoptosis, pH regulation, and cellular differentiation. Many studies are trying to find ways to reduce or stop HIF-1α to make treatments more effective. Khan et al. [57] studied the activity of doxorubicin hydrochloride-loaded oxygen nanobubbles on MDA-MB-231 breast cancer cells and HeLa pelvic cancer cells in vitro. The study demonstrated that oxygen nanobubbles reverse hypoxia and suppress HIF-1α activity in tumor cells.

Paclitaxel is one of several tubulin-targeting cytoskeletal drugs [58]. Mitotic spindle assembly, chromosome segregation, and cell division are all impaired in paclitaxel-treated cells; in contrast to different tubulin-targeting drugs that prevent microtubule assembly, such as colchicine, paclitaxel stabilizes the microtubule polymer and protects it from disassembly [59]. As a result, chromosomes are unable to achieve a metaphase spindle configuration. This process prevents mitosis from progressing, and prolonged mitotic checkpoint activation causes apoptosis or cell cycle reversion to the G0-phase without cell division [60]. 

Gastrin-releasing peptides (GRP) are regulatory human peptides that elicit gastrin release and regulate gastric acid secretion and enteric motor function [61]. The vagus nerve postganglionic fibers that innervate the bombesin/GRP neurons of the stomach release GRP, and this triggers the G cells to release gastrin. ProGRP is the most common, highly effective, and accurate biomarker for the diagnosis and treatment of small cell lung cancer (SCLC) [62]. Therefore, targeting ProGRP to stop SCLC growth could be used as a therapeutic treatment. Wang et al. [63] analyzed the efficiency of a paclitaxel-loaded nanobubble with pro-gastrin-releasing peptide against small cell lung cancer in vitro and in vivo. Paclitaxel targeting nanobubbles efficiently impeded the growth, migration, and infiltration of SCLC cells while inducing apoptosis in these cells.

**Figure 3:**
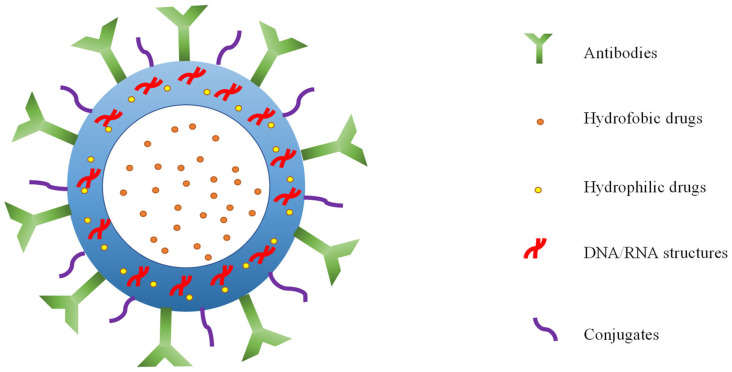
Modifications of nanobubbles.

By inhibiting oncogene expression, gene therapies may postpone tumor development. Neuroepithelial-transforming protein 1 (*NET-1*) gene, together with *NET-2* to *NET-7* genes, called *NET-x*, belongs to the tetraspan superfamily (*TM4SF*) and was discovered by Serru et al. [64] from the EST (Expressed Sequence Tags) database in the year 2000. Compared to normal tissue, hepatocellular carcinoma (HCC) showed an increased expression of the *NET-1* gene [65]. It has been shown that *NET-1* was strongly associated with pathological grading and clinical stages of HCC [66], making *NET-1* a potential therapeutic target for this disease. Consequently, *NET-1* may be an impactful target for gene therapy. Shang et al. [67] evaluated the therapeutic effect of nanobubbles conjugated with *NET-1* siRNA by shear wave elastography in vivo. *NET-1* gene therapy significantly delayed the tumor size growth compared to the control and the blank group, and its expression was significantly lower than these two groups. Modifications of nanobubbles against cancer are shown in Figure 3.

The scientific basis of sonodynamic therapy (SDT) uses low-intensity ultrasound, combined with oxygen, and an enhancement drug to generate reactive oxygen species (ROS) [68]. SDT is an extension of photodynamic therapy (PDT). PDT alone has some limitations, as cure rates are reported for very superficial lesions (tumor thickness 2–3 mm). A low tissue-penetrating depth of light is not effective for deep tumors such as hepatocellular carcinoma [69]. To avoid phototoxicity, patients should be kept out of direct sunlight after receiving a photosensitizer injection [70]. On the other hand, ultrasound is a safe and reliable imaging technology that penetrates deeply into human tissue. A combination of the two (sonodynamic) provides low toxicity, noninvasiveness, and high repeatability [71]. 

Hematoporphyrin monomethyl ether (HMME) is a porphyrin-related agent used as the sonosensitizer in cancer therapy. HMME is prepared from hemin as a derivative of protoporphyrin IX, where the two vinyl groups have been hydrated (converted to alcohol) [72]. HMME consists of two monomer porphyrins, namely, 3-(1-methyloxyethyl)-8-(1-hydroxyethyl) deuteropor-phyrin IX and 8-(1-methyloxyethyl)-3-(1-hydroxyethyl) deuteroporphyrin IX that are mutually locational isomers [73]. As HMME is not very stable or soluble in water, it limits the extent to which the body can absorb it [74]. When combined with ultrasound, it has a strong effect on cells, so it can be sucessfully used in clinics [75]. Research has demonstrated that HMME-PDT can cause cell death through both necrosis and apoptosis. The use of sodium azide (a singlet oxygen quencher) or D-mannitol (a hydroxyl radical scavenger) has been shown to protect HeLa cells from the apoptosis and necrosis induced by HMME-PDT. This indicates that reactive oxygen species (ROS), such as singlet oxygen and the hydroxyl radical, play a crucial role in the cell death induced by HMME-PDT. Additionally, the use of sodium azide or D-mannitol has been found to inhibit the HMME-PDT-induced increase in intracellular calcium ([Ca^2+^]i). The inhibition of Cytochrome C (Cyto C) release from mitochondria and the activation of Caspase-3 following HMME-PDT by BAPTA/AM, an intracellular calcium chelator, suggests that the reactive oxygen species (ROS) produced in HeLa cells during HMME-PDT-induced apoptosis may be linked to an elevation in intracellular calcium levels ([Ca^2+^]i). This rise in calcium levels may then trigger the release of Cyto C and the activation of Caspase-3, initiating the subsequent late stages of apoptosis [76]. 

Lonidamine (LND) is a derivative of indazole-3-carboxylic acid, which for a long time, has been known to inhibit aerobic glycolysis in cancer cells. It seems to enhance aerobic glycolysis in normal cells but suppresses glycolysis in cancer cells and is found to have anti-tumor activity by acting on tumor mitochondria [77,78]. Lonidamine is a small molecule that can be administered orally. It works by inhibiting glycolysis through the inactivation of hexokinase, an enzyme that catalyzes the first step of glycolysis, which is the breakdown of glucose. Research has shown that mitochondria and the hexokinase bound to them play a crucial role in causing apoptosis, a process of programmed cell death. Substances that directly affect the mitochondria may cause apoptosis. In laboratory studies, lonidamine has been found to exhibit the characteristics of apoptosis, including mitochondrial membrane depolarization, the release of cytochrome C, the externalization of phosphatidylserine, and DNA fragmentation [79,80]. A recent study has examined the potential of combining LND with chemotherapeutic agents or physical therapies. The findings indicate that the use of the nanometer system, encapsulated with LND, could significantly enhance the anticancer effects of drugs or the therapeutic efficacy of physical therapies. This approach shows promise in improving tumor targeting and could lead to more effective cancer treatments in the future. Shang et al. [81] demonstrated that HMME-LND@C3F8-NBs conjugated with low-frequency ultrasound inhibited the development of hepatocellular carcinoma cells, generated ROS, and significantly reduced the mitochondrial membrane potential, promoting HCC cells apoptosis effectively. 

Nuclear factor erythroid 2 (NFE2)-related factor 2 (NFE2L2, or NRF2) is a pivotal transcription factor that exerts a main influence on the expression of antioxidant genes. Its role in maintaining redox balance is of great importance, particularly in the context of cancer cells. The activation of NRF2 orchestrates a wide spectrum of cancer markers, including the regulation of metabolism, modulation of cancer stem cell features, tumor aggressiveness, facilitation of invasion, and promotion of metastasis formation [82]. In conditions of low oxidative stress, Nrf2 is sequestered in the cytosol through its interaction with the inhibitor Keap1 (Kelch-like ECH-associated protein), which mediates Nrf2 ubiquitination and subsequent proteasomal degradation. However, when oxidative stress occurs, the oxidation of Keap1’s cysteine residues induces a conformational change, leading to the liberation of Nrf2. Consequently, the unleashed Nrf2 translocates to the nucleus and binds to antioxidant response element (*ARE*) sequences within the promoters of genes encoding various antioxidant enzymes, including Heme oxygenase-1 (HO-1) and glutathione-S-transferase (GST). Moreover, it regulates genes involved in glutathione (GSH) synthesis, such as -glutamate-cysteine ligase (GCL), which is important in the biosynthesis of GSH. The multifaceted impact of NRF2 underlines its significance in cancer biology and positions it as a compelling target for therapeutic intervention. Its involvement in redox homeostasis, cancer cell survival, and metastatic progression makes NRF2 an interesting element for further search for novel anticancer strategies [83,84]. Nevertheless, an accumulating body of evidence shows that the cancer upregulation of Nrf2 affects cell proliferation, epithelialmesenchymal transition (EMT), migration, invasion, and angiogenesis as well as the chemo- and radioresistance of various malignant tumors, including melanoma [85,86,87]. Research on malignant melanoma samples has found that Nrf2 expression is linked to deeper Breslow, an invasive phenotype, nodular growth, and poorer survival [88]. Other data have shown that Nrf2 is excessively activated in chemoresistant melanoma cells, and reducing its expression, including through the use of small interfering RNA (siRNA), has improved melanoma sensitivity to standard drugs [89]. Interestingly, Nrf2 activation is also implicated in acquired resistance to targeted molecular therapy, such as anti-BRAF treatment [90], and inhibiting Nrf2 can help overcome melanoma radioresistance [91]. Given these findings, Nrf2 holds promise as a target for predicting tumor sensitivity to chemotherapy, and inhibiting it could help overcome resistance to chemotherapy and radiotherapy [92]. Using a specific siRNA against the *NFE2L2* gene can enhance the specificity of this approach. siRNAs are small double-stranded RNA molecules, typically 21–24 nucleotides long, that have been widely used to inhibit tumor-promoting factors and reverse chemoresistance [93,94]. siRNAs are difficult to use in clinical treatments because they are quickly enzymatically broken down in the body, quickly removed by the kidneys, and not taken up by cells because they are hydrophilic, large, and negatively charged. They also has to overcome several biological barriers to reach cells [95,96]. Argenziano et al. [92] created a new type of nanobubble for delivering siRNA against Nrf2 with ultrasound. It seems that siNrf2-NBs downregulated the target gene in M14 melanoma cells, making the cells more sensitive to cisplatin treatment. The combination with ultrasound increased the nanobubble’s ability to enter cells and deliver the siRNA.

**Table 1:** In vitro/in vivo results of nanobubble modifications against different cancers.

Drug/Oxygen/Antibody Delivery Technique	Analyzed Material	Measurement Techniques	Outcomes	References
Chitosan nanobubbles with a perfluoropropane core and doxorubicin hydrochloride combined with ultrasound (DOX-NBs + US)	MCF-7 cells (Michigan Cancer Foundation-7 cells), breast cancer cells, in vitro	Confocal images with Annexin V staining, flow cytometry	Increased percentage of apoptotic cells compared to a group with free DOX (45.7 ± 1.1% vs. 4.4 ± 0.9%, *p* < 0.01)	[54]
Doxorubicin hydrochloride-loaded oxygen core nanobubbles with phospholipid shell	MDA-MB-231 (MD Anderson-Metastatic Breast-231 cancer cells), HeLa cervical cancer cells, in vitro	Reactive oxygen species assays (ROS), confocal images, fluorescence, DAPI staining	Reversed hypoxia Increased generation of ROS Suppression of hypoxia-inducible factor-1 alpha activity in tumor cells Increased drug effects	[57]
Paclitaxel-loaded nanobubbles with anti-pro-gastrin-releasing peptide antibody	SCLC (small cell lung cancer), H446 lung cancer cells, in vitro, in vivo	Reverse-transcription polymerase chain reaction (RT-PCR), Western blot, immunohistochemical detection, CCk-8 assay, flow cytometry, cell scratch test, tumor-burden nude mice models	Inhibited SCLC cell proliferation, migration, and promotion of cell apoptosis (28.28 ± 4.2%) Decreased expression of Bcl-2, surviving, CDK2, MMP-2 (*p* < 0.01), increased expression of caspase-3 and Rb (*p* < 0.05) Lower tumor weight than the control group (*p* = 0.001)	[63]
Targeting nanobubbles conjugated with *NET-1* (Neuroepithelial cell-transforming gene 1) siRNA by shear wave elastography	Hepatocellular carcinoma (HepG2)-bearing mice model, in vivo	Ultrasound, shear wave elastography (SWE), immunohistochemical analysis	NET-1 gene (treatment group) significantly delayed (*p* < 0.0001) the growth of tumor size compared to the other two groups (*p* < 0.0001) The expression of NET-1 protein was significantly lower in the negative control and blank groups	[67]
Hematoporphyrin monomethyl ether (HMME) with Lonidamine(LND) liposome nanobubbles (NBs) with perfluorocarbone core in combination with US (C_3_F_8_) HMME-LND@C3F8-NBs	HCC (Hepatocellular carcinoma) Huh7 and HepG2 cancer cell lines, in vitro	CCk-8 assay, intracellular ROS generation detection and mitochondrial membrane potential assay, cell apoptosis assay, measurement of whole transcriptome library, quantitative reverse transcription-polymerase chain reaction (qRT-PCR)	NBs combined with HMME and LND decreased cell viability more significantly than LND alone, HMME alone, and NBs combined with HMME	[81]
Chitosan-shelled nanobubble for the delivery of siRNA against Nrf2 in combination with US	M14 melanoma cancer cells, in vitro	Fluorescence microscopy, viability analysis, Western blot, cytofluorometric evaluation	siRNA was successfully loaded in NBs, reaching an encapsulation efficiency of about 90% siNrf2-NBs downregulated the target gene in M14 cells, sensitizing the resistant melanoma cells to the cisplatin treatment The combination with ultrasound favored NB cell uptake and transfection efficiency	[92]

## 5. Conclusions and Future Perspectives in Cancer Treatment

The scientific study of nanobubbles has advanced significantly in recent years, particularly regarding anticancer treatment. In vivo and in vitro studies on cancer confirm nanobubble efficacy and good blood capillary perfusion; however, there is a need to define its safety and side effects in clinical trials. Many studies have demonstrated that conjugate nanobubbles can be a more effective therapeutic tool than conventional methods. On the other hand, the clinicaltrials.gov portal reports that no clinical studies involving cancer patients are currently underway. Studies on people concern malaria diagnosis, hypoxia, pulmonary fibrosis, or Amyotrophic Lateral Sclerosis (ALS). It is interesting to note that nanobubbles present possibilities for preclinical research on treatment optimizations in addition to serving as a tracking tool for cancer immunotherapy. Jiang et al. [97] studied the post-infusion monitoring of natural killer (NK) cells via ultrasound imaging in vivo. Nanobubbles served as contrast agents specific to NK cells in monotherapy and synergic treatment with cytokines. After co-treatment with interleukin-2 (IL)-2, the group observed that NK cells reached the tumor site after 3 h post-infusion. These infused immune cells’ trafficking, infiltration, persistence, and anti-tumor activities in the tumor microenvironment (TME) provide important data for evaluating the therapeutic effects after treatment and creating adjuvant strategies to maximize those effects. Overall, nanobubbles are the newest branch of nanotechnology and can be used in cancer therapy because they work synergistically with conventional therapy [98] and at the same time, can be complementary to other nanotechnologies (e.g., nanogels, nanotubes, nanoparticles); however, this should be confirmed at the clinical level.

## Data Availability

Not applicable.

## Abbreviations

AFM	atomic force microscopy.
ALS	amyotrophic lateral sclerosis.
ARE	antioxidant response element.
ASRs	age-specific rates.
ATF4	activating transcription factor 4.
BAPTA	1:2-bis(o-aminophenoxy)ethane-N,N,N′,N′-tetraacetic acid.
BBB	blood-brain barrier.
BNBs	bulk nanobubbles.
BRAF	activating mutations in cytoplasmic serine/threonine kinase B-Raf gene.
CCk-8	cell counting kit-8.
DAPI	4′:6-diamidino-2-phenylindole dihydrochloride.
DOX	doxorubicin.
EMT	epithelialmesenchymal transition.
GCL	glutamate cysteine ligase
GRP	gastrin–releasing peptide.
GSH	glutathione.
GST	glutathione-s-transferase.
H446	lung cancer cell line.
HCC	hepatocellular carcinoma.
HeLa	cervical cancer cell line.
HepG2	hepatocellular carcinoma cell line.
HIF1α	hypoxia-inducible factor.
HMME	hematoporphyrin monomethyl ether.
HMME-LND@C3F8-NBs	hematoporphyrin monomethyl ether–lonidamine perflurorocarbon nannobubbles.
HMME-PDT	hematoporphyrin monomethyl ether photodynamic therapy.
HO-1	heme oxygenase-1.
Huh7	hepatocellular carcinoma cell line.
IL-2	interleukin-2.
ISO	international organization for standardization.
Keap1	kelch-like ECH-associated protein.
LND	lonidamine.
M14	melanoma cancer cell line.
MCF-7	breast cancer cell line.
MDA-MB-231	breast cancer cell line.
MDR	multidrug resistance.
mTOR	mammalian target of rapamycin.
NB–nanobubble.	
*NET-1*	neuroepithelial-transforming protein 1 gene.
*NET-2*	neuroepithelial-transforming protein 2 gene.
*NET-7*	neuroepithelial-transforming protein 7 gene.
*NET-x*	neuroepithelial-transforming protein gene family.
NK	natural killer cells.
NRF2, Nrf2	nuclear factor erythroid 2 protein.
*NFE2L2*	NFE2 Like BZIP transcription factor 2 gene.
PDT	photodynamic therapy.
PLA	poly(lactic acid).
PLGA	poly(lactic-*co*-glycolic acid).
ProGRP	pro-gastrin-releasing peptide.
qRT-PCR	quantitative reverse transcription-polymerase chain reaction.
ROS	reactive oxygen species.
RT-PCR	reverse-transcripton polymerase chain reaction.
SCLC	small cell lung cancer.
SDT	sonodynamic therapy.
siRNA	small interfering RNA.
SNBs	surface nanobubbles.
SREBP2	sterol regulatory element-binding protein 2.
SWE	shear wave elastography.
*TM4SF*	tetraspan superfamily gene.
TME	tumor microenvironment.
US	ultrasound.
